# Intraoperative hand strength as an indicator of consciousness during awake craniotomy: a prospective, observational study

**DOI:** 10.1038/s41598-021-04026-9

**Published:** 2022-01-07

**Authors:** Chinatsu Umaba, Yohei Mineharu, Nan Liang, Toshiyuki Mizota, Rie Yamawaki, Masaya Ueda, Yukihiro Yamao, Manabu Nankaku, Susumu Miyamoto, Shuichi Matsuda, Hiroyuki Inadomi, Yoshiki Arakawa

**Affiliations:** 1 Rehabilitation Unit, Kyoto University Hospital, Kyoto, Japan; 2 Department of Human Health Sciences, Kyoto University Graduate School of Medicine, Kyoto, Japan; 3 Department of Neurosurgery, Kyoto University Graduate School of Medicine, 54 Shogoin Kawahara-cho, Sakyo-ku, Kyoto, 606-8507 Japan; 4 Department of Artificial Intelligence in Healthcare and Medicine, Kyoto University Graduate School of Medicine, Kyoto, Japan; 5 Department of Anesthesia, Kyoto University Graduate School of Medicine, Kyoto, Japan; 6 Department of Orthopedic Surgery, Kyoto University Graduate School of Medicine, Kyoto, Japan

**Keywords:** Neurology, Neurological manifestations

## Abstract

Awake craniotomy enables mapping and monitoring of brain functions. For successful procedures, rapid awakening and the precise evaluation of consciousness are required. A prospective, observational study conducted to test whether intraoperative hand strength could be a sensitive indicator of consciousness during the awake phase of awake craniotomy. Twenty-three patients who underwent awake craniotomy were included. Subtle changes of the level of consciousness were assessed by the Japan Coma Scale (JCS). The associations of hand strength on the unaffected side with the predicted plasma concentration (Cp) of propofol, the bispectral index (BIS), and the JCS were analyzed. Hand strength relative to the preoperative maximum hand strength on the unaffected side showed significant correlations with the Cp of propofol (ρ =  − 0.219, *p* = 0.007), the BIS (ρ = 0.259, *p* = 0.002), and the JCS (τ =  − 0.508, *p* = 0.001). Receiver operating characteristic curve analysis for discriminating JCS 0–1 and JCS ≥ 2 demonstrated that the area under the curve was 0.76 for hand strength, 0.78 for Cp of propofol, and 0.66 for BIS. With a cutoff value of 75% for hand strength, the sensitivity was 0.76, and the specificity was 0.67. These data demonstrated that hand strength is a useful indicator for assessing the intraoperative level of consciousness during awake craniotomy.

## Introduction

The use of intraoperative neuromonitoring during awake craniotomy prevents serious neurological sequelae and allows more extensive tumor resection^1,2^. Rapid awakening and maintenance of full consciousness after the cessation of general anesthesia are important for accurate monitoring of motor and language functions, which have a significant impact on the patient's quality of life after craniotomy^3^. Thus, proper monitoring of the level of consciousness is particularly important for successful procedures.

Target-controlled infusion (TCI) of propofol is used to achieve an adequate speed and quality of recovery of neurological functions from anesthesia^4,5^. Propofol has a short half-life and is rapidly metabolized to lower blood levels, making it a widely used drug in awake craniotomy. Changes in a patient's level of consciousness can be monitored by the bispectral index (BIS)^6,7^, which correlates well with the propofol concentration^8^, and is thus routinely used to monitor depth of anesthesia. The anesthesiologist determines whether the patient can be extubated and moved to the awake phase after confirming that the patient's BIS is elevated.

During awake craniotomy, patients may be somnolent or unable to fully follow verbal instructions, even though they have been awakened from anesthesia and extubated^9^. It has been shown that the return of BIS to preinduction values was associated with patients’ ability to perform intraoperative language testing^10^. However, during intraoperative monitoring, the presence of electrical artifacts reduces the reliability of the BIS. In addition, effective measurement of the BIS may not be possible if the BIS sensor cannot be placed on the forehead (e.g., bilateral frontal craniotomy). When reliable data from the BIS are not available to assess the patient's level of consciousness (e.g., electrophysiological artifact), other monitoring modalities are required.

Hand strength is routinely monitored as one of the important neurological functions (motor function). Because motor evoked potential (MEP) amplitude and latency were shown to be closely correlated with depth of anesthesia^11^, we expected that hand strength may also reflect the depth of anesthesia and the level of consciousness. If this were the case, we could monitor both motor function and arousal by one examination modality. Hand strength on the unaffected side is expected to represent arousal, and that on the affected side represents motor function. However, hand strength is often measured qualitatively by the examiner, and the relationship with the level of consciousness has not been well studied. In the present investigation, hand strength was assessed quantitatively by a hand dynamometer, and it was standardized by calculating percent strength relative to the preoperative maximum hand strength. Then, the aim was to test the hypothesis that hand strength on the unaffected side reflects the depth of anesthesia and can be used to monitor the level of consciousness.

Globally, the Glasgow Coma Scale (GCS) is most widely used to assess the level of consciousness, with a total score of 3–15: 1–4 for eye opening (E), 1–5 for verbal response (V), and 1–6 for best motor response (M)^12–14^. A higher score indicates better consciousness. In Japan, the Japan Coma Scale (JCS) is widely used together with the GCS (Table 1)^15^. The 1-digit code in the JCS consists of the scores of 3 (able to keep eyes open and obey, but unable to respond with names or places), 2 (residual disorientation), and 1 (mostly conscious). Zero represents clear consciousness. JCS 0 and 1 correspond to GCS 15, and JCS 2 and 3 correspond to GCS 14^16^, meaning that the JCS can discriminate subtle changes in consciousness after the patient opens his/her eyes and obeys verbal commands. During brain mapping of motor/language function or extracting the eloquent area, it is important that the patient is awake at the level of JCS 0 or 1. In patients immediately after extubation with a JCS of 2 or more, it is difficult to validly assess motor and language functions because of their slow reaction time^17^. Therefore, the level of consciousness required for continuous neuromonitoring was considered to be JCS 0 or 1. In addition, patients can sometimes open their eyes and obey a command immediately after extubation, but they are unable to speak^9,18,19^. The patients are regarded as JCS 3, but it is difficult to tell whether they are not fully awake or they are awake but unable to speak due to a seizure or insufficient recovery of language function due to remaining effects of anesthesia. Other limitations of the evaluation of consciousness by the coma scale were described by Guay et al.^20^. It allows only intermittent assessments of arousal. It provides only a categorical measure and may also yield equivocal responses. Moreover, it offers low time resolution, since repetition of the same questions in a short time period reduces the reliability of the response. In this context, additional modalities that can support the evaluation of the level of arousal are warranted. Hand strength may provide alternative measures of arousal with continuous values at a high time resolution. To evaluate the usefulness of hand strength on the unaffected side as an indicator of the intraoperative level of consciousness during awake craniotomy, its ability to discriminate between JCS 0–1 and JCS ≥ 2 was evaluated and compared with that of the BIS and other modalities.

**Table 1:** Japan Coma Scale for grading of impaired consciousness.

Grade	Consciousness Level
0	Fully conscious
**1-digit code**	The patient is awake without any stimuli, and is:
1	Almost fully conscious
2	Unable to recognize time, place, and person
3	Unable to recall name or date of birth
**2-digit code**	The patient can be aroused (then reverts to previous state after cessation of stimulation):
10	Easily by being spoken to (or is responsive with purposeful movements, phrases, or words)*
20	With loud voice or shaking of shoulders (or is almost always responsive to very simple words like yes or no, or to movement)*
30	Only by repeated mechanical stimuli
**3-digit code**	The patient cannot be aroused with any forceful mechanical stimuli, and:
100	Responds with movements to avoid the stimulus
200	Responds with slight movements including decerebrate and decorticate posture
300	Does not respond at all except for change in respiratory rhythm

*Criteria in parentheses are used in patients who cannot open their eyes for any reason. This table was quoted with modification from Ohta et al.^3^.

## Results

### Characteristics of the study population

Twenty-three patients were included in the analysis; their characteristics are shown in Table 2. The mean age of the patients was 49.6 ± 17.4 years, and 19 (82.6%) were male. The lesions were in the left hemisphere in 17 patients and in the right hemisphere in six patients. The mean hand strength of the unaffected side measured preoperatively was 34.2 ± 9.1 kg, and that of the affected side was 31.4 ± 9.4 kg. During the awake craniotomy, the mean time from cessation of anesthetic administration to extubation was 19.4 ± 7.9 min.

**Table 2:** Patients’ characteristics.

Patient no	Sex	Age (years)	Tumor location	Hemisphere	WHO grade	Diagnosis	Preoperative hand strength (kg)	
							Unaffected side	Affected side
1	M	49	Parietal	Left	IV	Glioblastoma	Not measured	41.1
2	F	18	Parietal	Left	I	Ganglioglioma	27.4	26.4
3	M	63	Temporal	Left	IV	Glioblastoma	Not measured	35.2
4	M	38	Insula	Left	IV	Giant cell glioblastoma	32.9	23.2
5	F	73	Frontal	Left	II	Oligodendroglioma	26.3	27.8
6	M	72	Temporal	Left	III	Anaplastic astrocytoma	28.1	30.3
7	M	25	Frontal	Left	II	Oligodendroglioma	42.6	45.9
8	M	63	Frontal	Left	–	Tufted angioma	27.3	32.6
9	M	34	Temporal	Left	IV	Glioblastoma	29.4	35.2
10	F	47	Parietal	Right	IV	Glioblastoma	14.6	17.6
11	M	33	Frontal	Left	III	Anaplastic oligodendroglioma	25.4	23.2
12	M	77	Frontal	Right	IV	Glioblastoma	32.6	31.4
13	M	70	Frontal	Right	IV	Glioblastoma	35.8	12.3
14	M	54	Frontal	Left	III	Anaplastic astrocytoma	37.8	37.9
15	M	48	Parietal	Left	II	Oligodendroglioma	48.0	46.0
16	M	56	Frontal	Right	II	Diffuse astrocytoma	44.2	39.6
17	M	43	Frontal	Right	III	Anaplastic astrocytoma	34.5	34.7
18	M	43	Frontal	Left	IV	Glioblastoma	47.0	36.2
19	M	54	Frontal	Left	II	Diffuse astrocytoma	47.3	42.7
20	F	62	Temporal	Left	–	Gliosis	20.8	20.5
21	M	66	Parietal	Right	IV	Glioblastoma	35.2	27.6
22	M	17	Parietal	Left	IV	Glioblastoma	38.9	17.0
23	M	36	Frontal	Left	III	Anaplastic astrocytoma	42.3	38.9

### Chronological changes in consciousness after extubation

**Figure 1:**
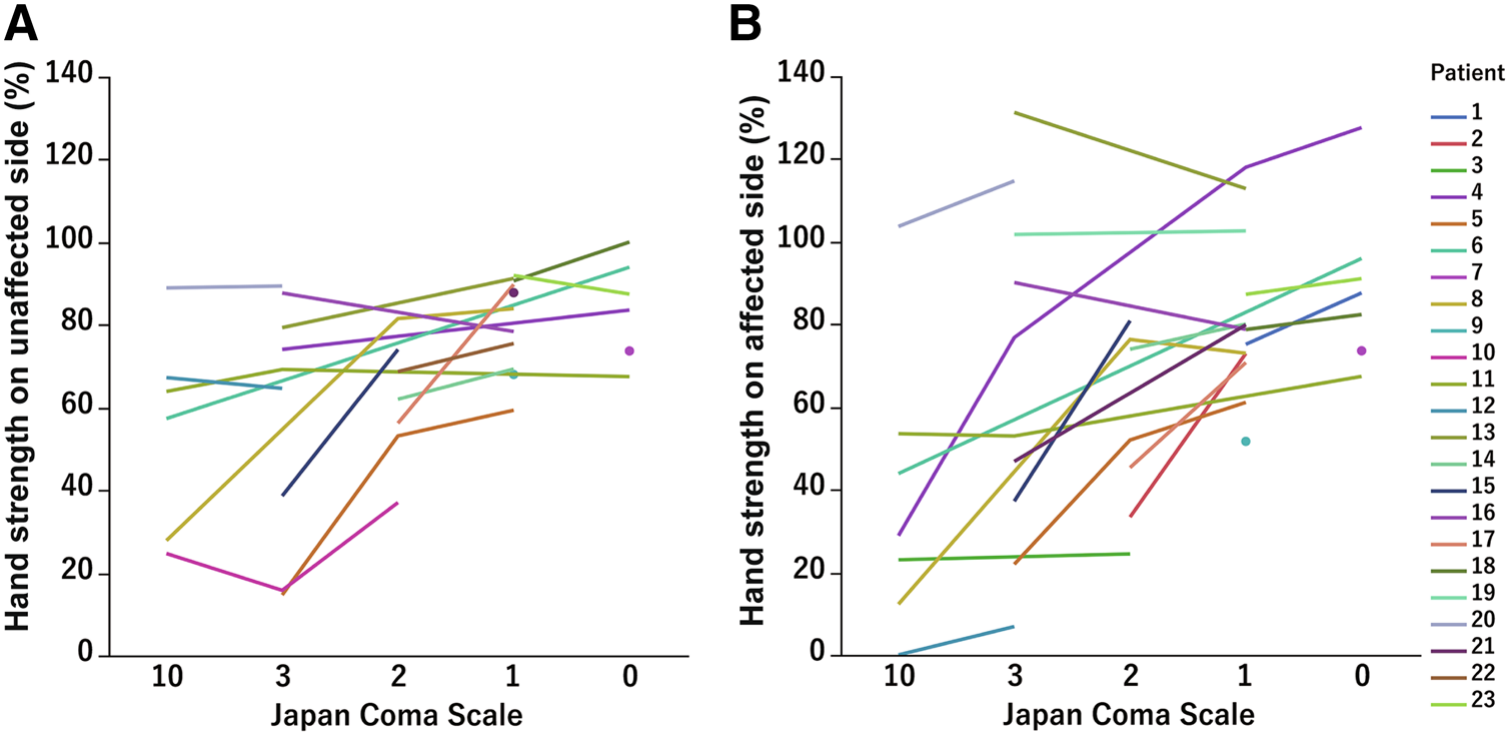
The representative values of hand strength tended to increase with an increase in the Japan Coma Scale (JCS). The line graphs show the change in hand strength at each JCS for each patient (**A**: unaffected hand, **B**: affected hand). Several plots show a patient that had no change in consciousness from the beginning to the end of the measurement of hand strength during the awake state.

The chronological changes in the patients' consciousness after extubation were evaluated using the JCS. Table 3 shows the mean (± standard deviation (SD)) minutes to reach each JCS level after extubation. The mean predicted plasma concentration (Cp) of propofol and the BIS score for each JCS are also shown. The number of patients at each JCS level was different because some did not become fully conscious, and others rapidly gained consciousness by skipping the level of JCS 10. Immediately after extubation, 18 patients were at JCS 10; 14.9 min were needed to reach JCS 3, 16.6 min to reach JCS 2, 26.4 min to reach JCS 1, and 30.0 min to reach JCS 0. The changes in consciousness level and hand strength for each patient are shown in Fig. 1. Percent hand strength relative to the preoperative maximum hand strength used as intraoperative hand strength in the analysis. The intraoperative hand strengths increased according to the improvement in the JCS in most cases, on both the unaffected and affected hands (Fig. 1).

**Table 3:** Time after extubation, predicted concentration of propofol, and the BIS at each JCS level.

N = 23	Time after extubation, min	Cp of propofol, μg/ml	BIS
	Mean (SD)	Mean (SD)	Mean (SD)
JCS 0 (n = 7)	30.0 (12.1)	0.58 (0.12)	87.0 (10.2)
JCS 1 (n = 19)	26.4 (16.3)	0.65 (0.11)	85.5 (8.6)
JCS 2 (n = 12)	16.6 (10.2)	0.75 (0.18)	85.7 (9.1)
JCS 3 (n = 14)	14.9 (14.7)	0.82 (0.28)	81.0 (13.2)
JCS 10 (n = 18)	1.0 (0.0)	0.99 (0.22)	80.9 (10.7)

*JCS* Japan Coma Scale, *Cp* predicted plasma concentration, *BIS* bispectral index, *SD* standard deviation.

### Correlations of hand strength, the Cp of propofol and the BIS with the JCS

**Figure 2:**
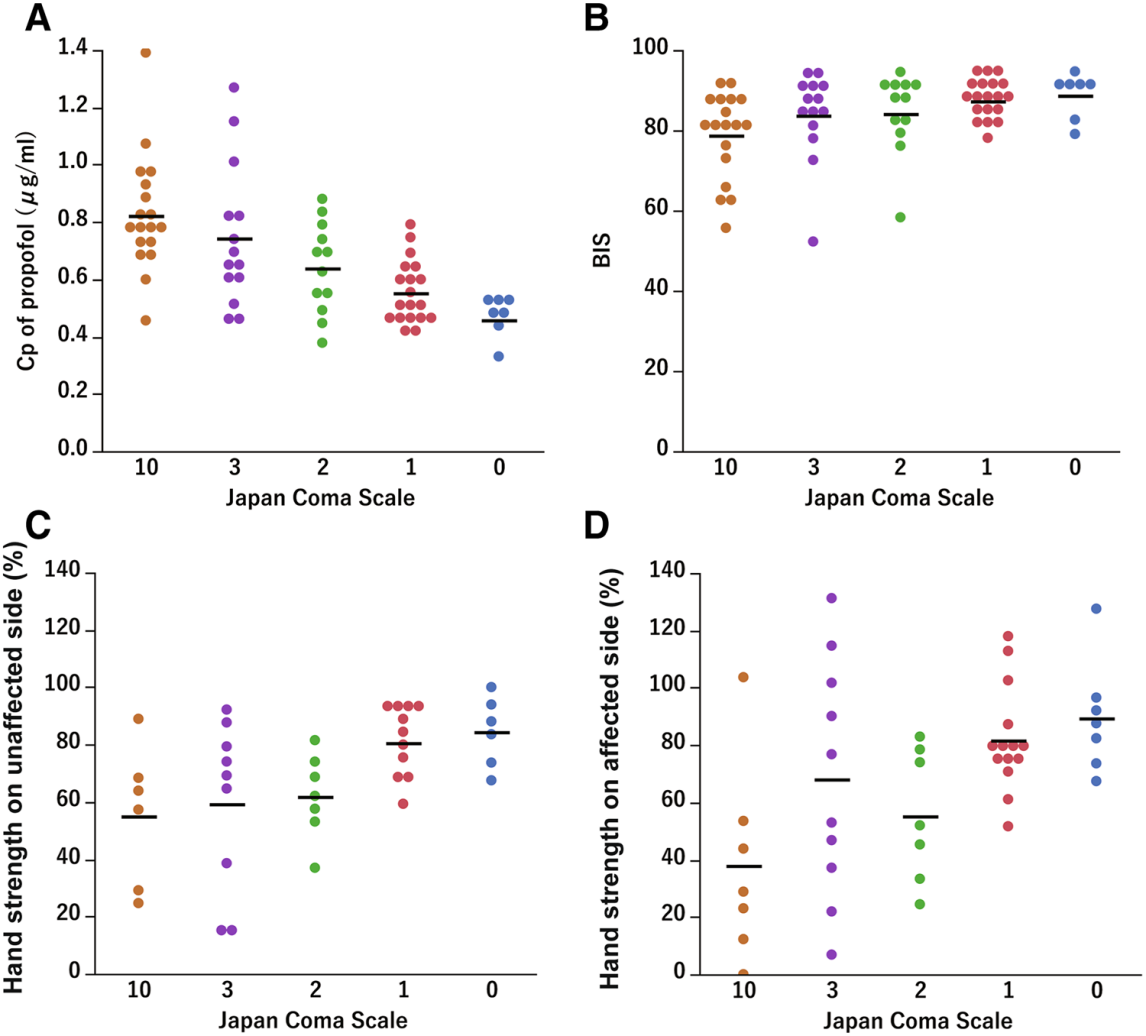
Plots showing the representative values averaged over the values for each Japan Coma Scale (JCS) period. The black bar shows the mean, and the values below the bars indicate the mean (standard deviation). Kendall’s rank correlation coefficient (τ) was used for analyses. (**A**) The predicted plasma concentration (Cp) of propofol decreases with the JCS (τ = 0.484, *p* < .0001). (**B**) The BIS increases with the JCS (τ =  − 0.269, *p* = 0.003). (**C**) Hand strength on the unaffected side increases with the JCS (τ =  − 0.508, *p* = 0.001). (**D**) Hand strength on the affected side increases with the JCS (τ =  − 0.351, *p* = 0.002).

The relationships between the JCS and the Cp of propofol, the BIS, and hand strength on both the affected and unaffected sides were evaluated using Kendall’s rank correlation coefficient. The Cp of propofol decreased from 0.82 ± 0.20 to 0.46 ± 0.06 µg/ml when the JCS decreased from 10 to 0, and this relationship showed a significant correlation (τ = 0.48, *p* < 0.01) (Fig. 2A). The BIS increased from 78.7 ± 10.8 to 88.7 ± 5.5 as the JCS decreased from 10 to 0, demonstrating a significant correlation (τ =  − 0.27, *p* < 0.01) (Fig. 2B). As the JCS decreased from 10 to 0, hand strength on the unaffected side increased from 55.0 ± 24.7% to 84.4 ± 12.2%, showing a significant correlation (τ =  − 0.51, *p* = 0.001) (Fig. 2C). Hand strength on the affected side also increased from 37.9 ± 34.2% to 89.4 ± 19.5%, and a significant correlation was observed (τ =  − 0.35, *p* = 0.002) (Fig. 2D). Deviation in the hand strength was greater on the affected side than the unaffected side. The coefficients of variation at JCS 10, 3, 2, 1, and 0 were 0.45, 0.49, 0.24, 0.14, and 0.14 for the unaffected hand, and 0.90, 0.61, 0.40, 0.23, and 0.22 for the affected hand, respectively.

### Hand strength was correlated with the Cp of propofol, reflecting depth of anesthesia

As shown above, the Cp of propofol, the BIS, and hand strength were significantly correlated with the JCS, indicating that they were good indicators of consciousness during awake craniotomy. Similarly, Spearman’s rank correlation coefficient analysis was conducted to determine the interactive effects of these parameters. A significant correlation was found between the Cp of propofol and hand strength on the unaffected side (Table 4; ρ =  − 0.219, *p* = 0.007), but not with hand strength on the affected side (ρ =  − 0.098, *p* = 0.151). This finding suggests that hand strength is also a good indicator of the depth of anesthesia. A significant correlation was also found between the BIS and hand strength on both sides (unaffected hand ρ = 0.259, *p* = 0.002; affected hand ρ = 0.277, *p* < 0.0001). A significant correlation was also found between the BIS and the Cp of propofol (ρ =  − 0.165, *p* < 0.001).

**Table 4:** Spearman's rank correlation coefficient (ρ).

	BIS		Hand strength on unaffected side		Hand strength on affected side	
	ρ	*p*	ρ	*p*	ρ	*p*
Predicted plasma concentration of propofol	− 0.165	< .0001*	− 0.219	0.007*	− 0.098	0.151
Bispectral index (BIS)			0.259	0.002*	0.277	< .0001*
Hand strength on unaffected side					0.713	< .0001*

*p < 0.05.

### Hand strength as an indicator of consciousness on Receiver operating characteristic (ROC) curve analysis

**Figure 3:**
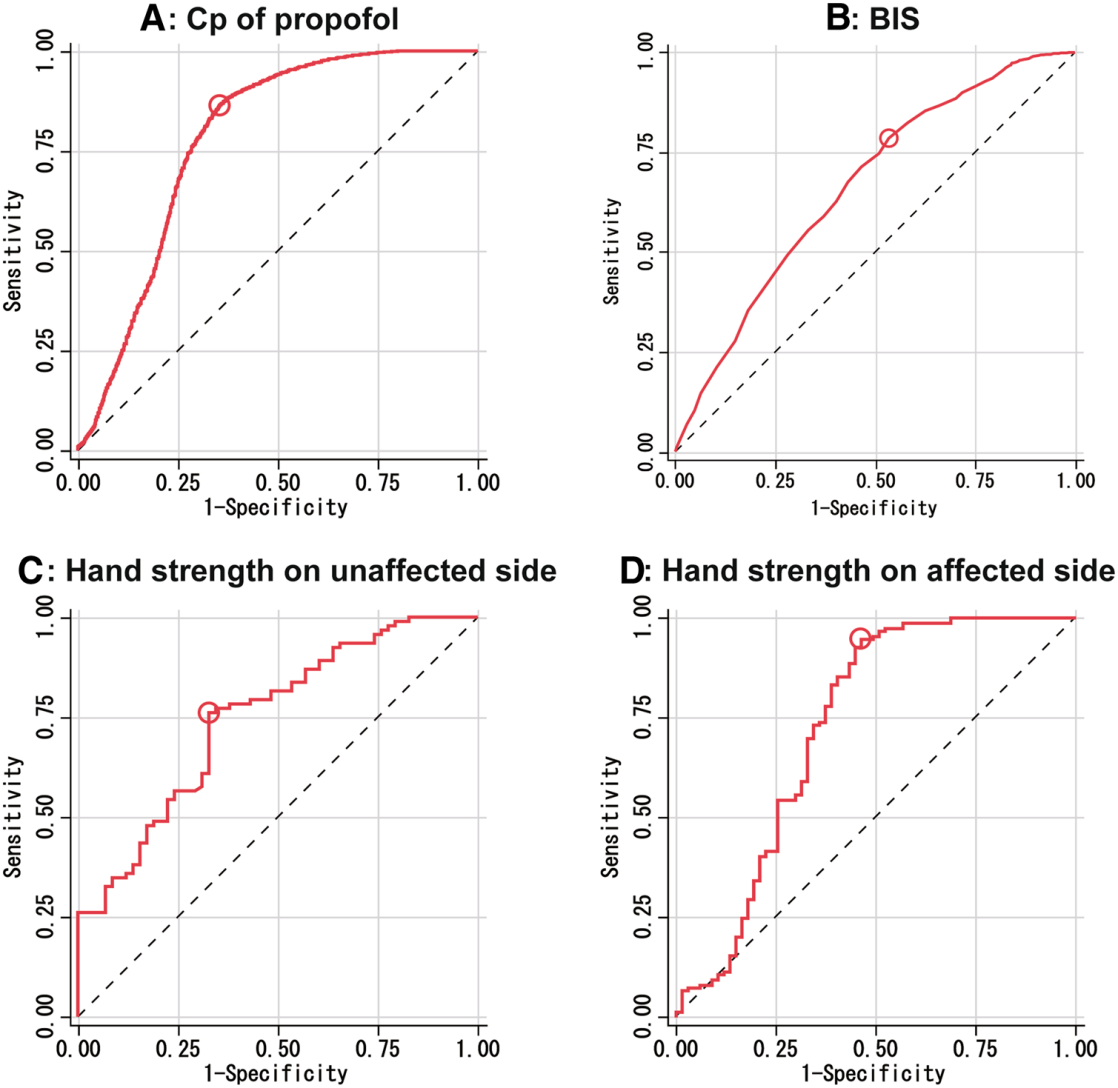
Receiver operating characteristic (ROC) analysis of the Japan Coma Scale (JCS). The red curves indicate JCS 0 or 1, (**A**) The predicted plasma concentration (Cp) shows an area under the curve (AUC) of 0.78 (*p* < .001). (**B**) The BIS shows an AUC of 0.66 (*p* < .001). (**C**) Hand strength on the unaffected side shows an AUC of 0.75 (*p* < .001). D: Hand strength on the affected side shows an AUC of 0.73 (*p* < .001).

To further evaluate the clinical significance of hand strength for assessment of the level of consciousness, ROC analysis was conducted (Fig. 3). The area under the curve (AUC) to discriminate whether the patient is in JCS level 0–1 or ≥ 2 was 0.75 for hand strength (*p* < 0.01). The sensitivity was 0.76, and the specificity was 0.67 at the cut-off value of 75% strength compared with preoperative maximum hand strength. The AUC of the affected hand was 0.73, and the sensitivity and specificity were 0.95 and 0.54, respectively, with a cut-off value of 56%. The AUC for the Cp of propofol was 0.78, the sensitivity was 0.86, and the specificity was 0.65 when the Cp of propofol was greater than or less than 0.61. In contrast, the AUC of the BIS was low (0.66), with sensitivity and specificity of 0.78 and 0.47, respectively. In terms of the AUC, moderate accuracy was obtained for both the unaffected- and affected-handgrip ratio. However, the specificity of the handgrip ratio of the affected hand was relatively low because of large individual differences due to intraoperative paralysis.

### Illustrative case

**Figure 4:**
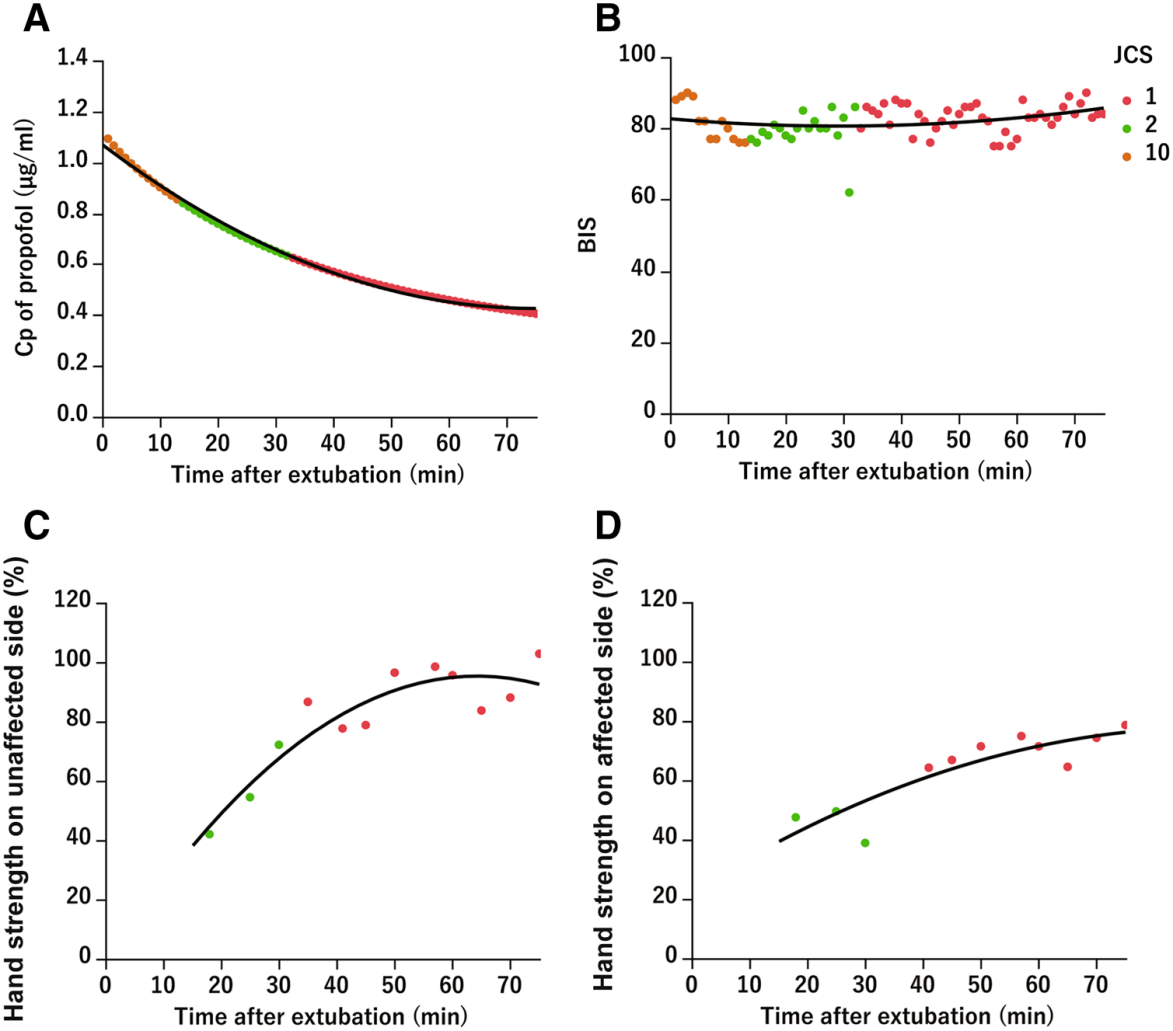
Illustrative case. Plots are each measurement in time after extubation of patient 17. The black curve shows the quadratic regression equation. The orange plot represents Japan Coma Scale (JCS) 10, the green is 2, and the red is 1. (**A**) The predicted concentration (Cp) of propofol decreases with time and the JCS. (**B**) The BIS changes very little with time and the JCS. (**C**) Hand strength on the unaffected side increases with time and approaches 100% with JCS 1. (**D**) Hand strength on the affected side increases with time but does not reach 100%.

Chronological changes of the indicators (Cp of propofol, BIS, hand strength) in patient No. 17 (43-year-old man) are shown in Fig. 4. The Cp of propofol decreased as time passed after extubation and was clearly correlated with the consciousness level. Specifically, the Cp was 1.01 at JCS 10, and it decreased to 0.62 at JCS 1 (33 min after extubation) (Fig. 4A). The BIS was over 80 immediately after extubation and remained between 70 and 90 afterwards (Fig. 4B). The hand strength of the unaffected hand increased sharply over time, from 40% at JCS 2 (20 min after extubation) to 80% at JCS 1 (30 min after extubation), showing a positive correlation with the level of consciousness. The hand strength remained high around 1.0 afterwards (Fig. 4C). The hand strength on the affected side also increased as time passed and showed a positive correlation with the level of consciousness, but the slope was shallow with a maximum of < 80% (Fig. 4D).

## Discussion

### Hand strength is useful as an intraoperative consciousness indicator during awake craniotomy

In the present study, it was demonstrated that hand strength relative to the preoperative maximum strength on the unaffected side showed a significant correlation with the Cp of propofol, which represents the depth of anesthesia. It was also correlated with the JCS. ROC curve analysis demonstrated that discrimination ability between JCS 0–1 and JCS ≥ 2 was satisfactory, with an AUC of 0.75 for the unaffected-hand grip strength, which was better than that of the BIS (0.66) and comparable to that of the Cp of propofol (0.78). With a cut-off value of 75% of hand strength, sensitivity was 0.76, and specificity was 0.67, indicating that hand strength is a good indicator of patients’ arousal and provides supportive information about whether the patient has reached a level of consciousness sufficient for monitoring motor and language functions^12,13,15^.

Relationship between hand strength and depth of anesthesia has previously been reported. A study showed that hand strength decreases in a dose-dependent manner when the Cp of propofol exceeds 0.8 μg/ml^21^. Another study showed that hand strength can be used to monitor the depth of anesthesia during induction with propofol^20^. Hand strength on the unaffected side is also a useful indicator of overall limb strength^22^. This is applicable to hemiplegic stroke patients^23^, suggesting that hand strength on the unaffected side can be used for patients with brain tumors, as well to assess overall limb strength. In the present study, unaffected hand strength of 75% or more relative to the preoperative level indicated a good level of consciousness. If this is not achieved, it will be difficult to determine whether the motor dysfunction is due to insufficient recovery from anesthesia or actual motor paralysis, making physiological monitoring unreliable. This would also be the case with verbal functions. Mapping and monitoring of brain functions should be initiated after we confirm sufficient recovery of hand strength, which reflects sufficient arousal and motor function.

The advantage of hand strength is that it can be measured with a small device that is a convenient tool for checking the results immediately. Therefore, hand strength can be measured repeatedly at a high time resolution and provides continuous values, making it a useful indicator for the assessment of the level of consciousness. Given that hand strength on the unaffected side can be used for monitoring the level of consciousness and that on the affected side can be used to monitor motor function, continuous measurement of hand strength would have substantial clinical benefits in awake craniotomy.

### Consciousness level of JCS1 or higher is required for language monitoring

Assessment of the level of consciousness includes use of the GCS and JCS, which are assessed from the patient's motor and verbal responses in general clinical practice. The Japanese Awake Craniotomy Guidelines do not specify a scale for assessing the level of consciousness at the beginning of neurological monitoring, especially language mapping^24^. In other studies, the level of consciousness to determine when language mapping can be initiated has not been clearly defined, since “mapping was initiated at the discretion of the neuropsychologist present”^10^ or no detailed description of the patient's level of consciousness is available^25,26^. Thus, the level of consciousness at which neurological mapping can be initiated has not been clearly defined. In other words, assessment of the level of consciousness during the awake phase has been subjective, and an objective assessment should be made immediately before starting the language task^27^.

When a patient breathes spontaneously and can follow simple verbal commands^21–23^, the patient can be extubated and shifted to the awake phase, even if not fully awake. However, to perform motor and verbal monitoring, nearly full consciousness is needed. To evaluate such subtle changes in consciousness, we used the JCS, which can evaluate consciousness in five linear steps (JCS 10, 3, 2, 1, and 0). JCS 3 and 2 indicate disorientation and do not indicate that the patient is ready to cooperate with monitoring. To accurately assess motor and language functions, reaching JCS1 (mostly conscious but slow to respond) or 0 (completely conscious) is needed. Thus, we considered that JCS 0–1 was a suitable condition for neurophysiological monitoring. Mapping and monitoring of higher brain functions during awake craniotomy have been recommended in the dominant hemisphere, as well as the non-dominant hemisphere^28^. In this context, hand strength is useful because we can measure it in both hands simultaneously.

A discrepancy between consciousness and hand strength can occur. When consciousness is disturbed despite a sufficient increase in hand strength, other adverse events may occur, such as seizures, discomfort, and/or language impairment. When the hand strength decreases despite a good level of consciousness, brain damage or ischemia may have occurred in the motor area or corticospinal tract. Therefore, a discrepancy between the level of consciousness and hand strength is helpful for early detection of adverse events during awake craniotomy.

### Reaching a sufficient consciousness level for language mapping takes time

In this study, an average of 19.4 min was required from anesthetic cessation to extubation, and an average of 26.4 min was required from extubation to JCS 1. Studies from Europe and the United States showed that the mean time from cessation of anesthesia to the initiation of mapping was 17.6 minutes^21^, and the median time from extubation to the initiation of mapping was 11 minutes^27^, both of which were shorter than those of the present study. This may be because of different pharmacokinetics between the Japanese and the European and US populations. The pharmacokinetic parameters used for propofol TCI have been validated in Japan^4,5^, but the times from anesthetic cessation to initial response are different between Europeans and Japanese (10.5 ± 6.37 min in Europeans, 20.1 ± 12.69 min in Japanese)^5,29^. Differences in height, weight, and other physical characteristics may have contributed to the ethnic differences in the time to full recovery of consciousness. To monitor language and higher brain functions accurately, the patient must be fully awake and cooperative. Patients should be assessed with a reliable measure of their level of consciousness to ensure that they are able to respond accurately and with appropriate speed to the task, and they should be allowed sufficient time to become fully conscious.

### Limitations

There are several limitations in the present study. First, the study population was small, and the results were not validated in either internal or external independent cohorts. Second, individuals who did not respond well to verbal commands due to somnolence were excluded from the analysis. Left-handed individuals were also excluded due to their history of dominant hand correction. Therefore, the results of the present study may not be applicable to all individuals. Third, inter-rater reliability analysis for the assessment of consciousness by the JCS was not performed. Fourth, multiple analyses were performed to show the robustness of hand strength to predict the level of consciousness, but this is susceptible to multiple testing error. However, the positive correlations were confirmed in all of the analyses, and therefore, multiplicity of analyses is unlikely to affect the interpretation of the results of the present study. Last, the correlation between the success rate of neuromonitoring and the recovery of hand strength was not examined. Further study is needed to address these limitations.

## Conclusions

Hand strength on the unaffected side was significantly correlated with the Cp of propofol and the BIS, supporting our assumption that hand strength is correlated with depth of anesthesia. It also showed a significant correlation with the JCS, and ROC curve analysis showed that JCS 0–1 and JCS ≥ 2 were efficiently distinguished by hand strength with a cutoff value of 75%. Routine measurement of hand strength on the unaffected side is easy and reliable. Thus, hand strength appears to be a good indicator of the level of consciousness during awake craniotomy. Further study is needed to validate these results and to test the generalizability to a larger population, including left-handed individuals.

## Methods

### Ethics approval and consent to participate

This study was approved by the Ethics Committee of the Graduate School of Medicine and Faculty of Medicine, Kyoto University, Japan (R1843-1). Written, informed consent was obtained preoperatively from all participants. All procedures were performed in accordance with relevant named guidelines and regulations including the Declaration of Helsinki and Ethical Guidelines for Medical and Biological Research Involving Human Subjects in Japan.

### Study population

The study population was prospectively collected from February 2020. Consecutive patients who underwent awake craniotomy at our hospital with a diagnosis of any brain tumor were included in the study. Thirty-two patients fulfilled the criteria between February 2020 and April 2021, and all of them gave their informed consent. According to the exclusion criteria, nine were excluded: 1) two patients were left-handed, 2) four patients were unable to communicate due to intraoperative seizures or delirium, 3) three patients had poor BIS measurements, the primary parameter of this study. Accordingly, 23 patients were finally analyzed in the present study.

### Anesthesia and the predicted concentration of propofol in the blood

Anesthesia during the awake craniotomy through all of the asleep-awake-awake phase was conducted based on a standardized institutional protocol, which was reported previously^30^. Anesthesia is induced and maintained by continuous infusion of propofol and remifentanil; muscle relaxants were not administered. In the analysis, the Cp of propofol in the blood was calculated from the propofol dosage in anesthesia records using Marsh’s pharmacokinetic model^31^.

### BIS monitoring

The BIS has routinely been used to monitor the depth of anesthesia since it was introduced by Aspect Medical Systems, Inc. in 1994^7^. Patch electrodes were positioned from the contra- and ipsilateral nasal bones to the ipsilateral zygomatic bone to avoid any noise due to craniotomy. The BIS was recorded before the craniotomy and throughout the operating procedure. The BIS score represents the electroencephalography activity during the immediately previous 60 s, and it was automatically calculated and recorded every 5 s as a unitless whole number between 0 and 100. In this study, the BIS values were stored in the surgical record every minute.

### Japan Coma Scale (JCS) assessments

The JCS levels are shown in Table 1^15^. During the awake phase of craniotomy, immediately after extubation, most patients show a level of consciousness that allows them to open their eyes in response to a call, understand verbal commands, and perform automatic movements (JCS: 10, GCS: E3V4M6; total score 13). If the eyes can be kept open, the JCS becomes a single digit, and the GCS becomes E4V4M6 (total score 14). Changes leading to better consciousness are judged by the verbal response, which is divided into two levels (E4V4M6; total score 14 or E4V5M6; total score 15) based on the presence or absence of confused speech in the GCS^16^, and four levels (3, 2, 1, 0) based on the presence or absence of disorientation and delayed response in the JCS (Table 1). In this study, level of consciousness of patients was assessed using the JCS, which can distinguish the level of consciousness in the awake phase more precisely. The evaluation was done by evaluators who knew the patient's condition before surgery. In order to avoid interactions, the evaluators of hand strength and those of the JCS were different.

### Measurement of hand strength

A hand dynamometer (MG4800, Charder Medical, Taiwan) was used to measure hand strength preoperatively and intraoperatively. Measurements were taken with both unaffected and affected hands. The affected hand was defined as the hand on the opposite side of the brain lesion. For preoperative measurements, hand strength was measured three times using the Max mode of the hand dynamometer on the day before surgery, and the average of these measurements was used as the preoperative value shown in Table 2. The evaluator encouraged the patient to exert maximum hand strength and instructed him/her to continue exerting hand strength for about 5 s. The measurement was performed in the supine position (same as the surgical position), with the elbow extended and the forearm and wrist in the intermediate position. This position is reproducible compared with the normal sitting position to evaluate hand strength^32^. To exclude the weight of the hand dynamometer, the dynamometer was placed on the operating table. For intraoperative measurements, hand strength during the awake state was measured intermittently with an interval of approximately 5 min to avoid the effects of muscle fatigue, and measurement was started after confirming that the patient had no nausea or delirium. The limb position and the settings of the hand dynamometer were performed under the same conditions as preoperative measurement. Intraoperative hand strength was presented as a percentage of the preoperative hand strength and used in the analysis.

### Statistical analysis

The continuous variables used in this study were the Cp of propofol, the BIS, and percentages of hand strength on the unaffected and affected sides; the JCS was treated as a categorical variable. The mean ± SD was used for descriptive statistics of continuous variables. The normal distribution was tested by the Shapiro–Wilk test. Since all variables were confirmed to not be normally distributed, a nonparametric approach was chosen for further analyses. Spearman's rank correlation coefficient was used to examine correlations among continuous variables (BIS, Cp of propofol, and hand strength), and Kendall's rank correlation coefficient was used to examine correlations between categorical variables and continuous variables (JCS and Cp of propofol, BIS, and grip strength). ROC analysis was used to evaluate the discrimination capacity between JCS 0–1 and JCS ≥ 2. To accurately show changes in the level of consciousness and to exclude the effects of noise and outliers in the data, Kendall's correlation analysis was performed using representative values of the Cp of propofol, the BIS, and hand strength, which were averaged for each level of JCS. All data were collected 1–75 min after extubation. All statistical analyses were performed using JMP Pro 15.2.0., and *p* < 0.05 was considered significant.

## Data Availability

The datasets used and/or analyzed during the current study are available from the corresponding author on reasonable request.

## Abbreviations

AUC: Area under the curve
BIS: Bispectral index
Cp: Predicted plasma concentration
GCS: Glasgow Coma Scale
JCS: Japan Coma Scale
MEP: Motor-evoked potential
ROC: Receiver operating characteristic
SD: Standard deviation
TCI: Target-controlled infusion
